# Multidisciplinary management of intracranial migration of a fractured dental needle through the foramen ovale: a case report

**DOI:** 10.1093/jscr/rjaf662

**Published:** 2025-09-23

**Authors:** Josep Rubio-Palau, Ferran Escrigas-Cañameras, Adaia Valls-Ontañon, Albert Malet-Contreras, Maria Victoria Becerra-Castro, Jordi Muchart-López, José Hinojosa-Bernal

**Affiliations:** Maxillofacial Surgery Unit, Sant Joan de Déu Hospital (SJD), Passeig Sant Joan de Déu 2, 08950, Esplugues de Llobregat, Spain; Plastic, Reconstructive and Aesthetic Surgery Department, Hospital Clínic de Barcelona (HCB), Carrer Villarroel 170, 08011, Barcelona, Spain; Maxillofacial Surgery Unit, Sant Joan de Déu Hospital (SJD), Passeig Sant Joan de Déu 2, 08950, Esplugues de Llobregat, Spain; Maxillofacial Surgery Unit, Sant Joan de Déu Hospital (SJD), Passeig Sant Joan de Déu 2, 08950, Esplugues de Llobregat, Spain; Neurosurgery Department, Sant Joan de Déu Hospital (SJD), Passeig Sant Joan de Déu 2, 08950, Esplugues de Llobregat, Spain; Radiology Department, Sant Joan de Déu Hospital (SJD), Passeig Sant Joan de Déu 2, 08950, Esplugues de Llobregat, Spain; Neurosurgery Department, Sant Joan de Déu Hospital (SJD), Passeig Sant Joan de Déu 2, 08950, Esplugues de Llobregat, Spain

**Keywords:** oral surgery, dental education, nerve block, needle breakage, needle migration, neuronavigation

## Abstract

Local anaesthesia and nerve block are common practices in dental therapy. Although they are associated with low rate of complications, local events such as needle fracture could entail life threatening consequences. We present a case of a needle fracture in a 4 year old girl who underwent a minor dental procedure. The needle migrated intracranially through the foramen ovale. She required a multidisciplinary management with a final neurosurgical intervention in order to remove it. In addition to an initial diagnostic computerized tomography (CT), it was of utmost importance to have access to an intraoperative imaging tools (preferably low radiation CT in paediatric population) to monitor the potential risk of needle migration. This report demonstrates that a broken needle may migrate intracranially, among other complications, underscoring the importance of managing these cases in a tertiary referral hospital with the appropriate equipment and specialists.

## Introduction

Local anaesthesia and nerve blocks are common practices in dental therapy [1]. Although they are associated with a low rate of complications (around 9%), potential risks include systemic reactions such as allergies, collapse, and hypertension, as well as local events like pain, haemorrhage, infection, soft tissue damage, nerve disorders, and needle fracture. The latter can entail potentially life-threatening consequences by damaging adjacent vital anatomical structures [2]. It is important to note that the vast majority of fractured needles are located in the pterygomandibular space, with a significant rate of migration (close to 10%) to other regions, such as the skull base, cervical region, or auricular area [3]. Migration into the cranial vault is rare due to the small size of the skull base foramina [4]. In such cases, symptoms usually include pain and tenderness, though other less common symptoms such as trismus, swelling, dysphagia, foreign body sensation, paraesthesia, or hearing loss may occur. Therefore, removal of the broken needle fragment is usually recommended as soon as possible, and a computerized tomography (CT) scan is the proper diagnostic tool for three-dimensional localization of a broken needle [5].

This case report presents a rare instance of intracranial migration of an anaesthetic injection needle through the foramen ovale.

## Case report

**Figure 1:**
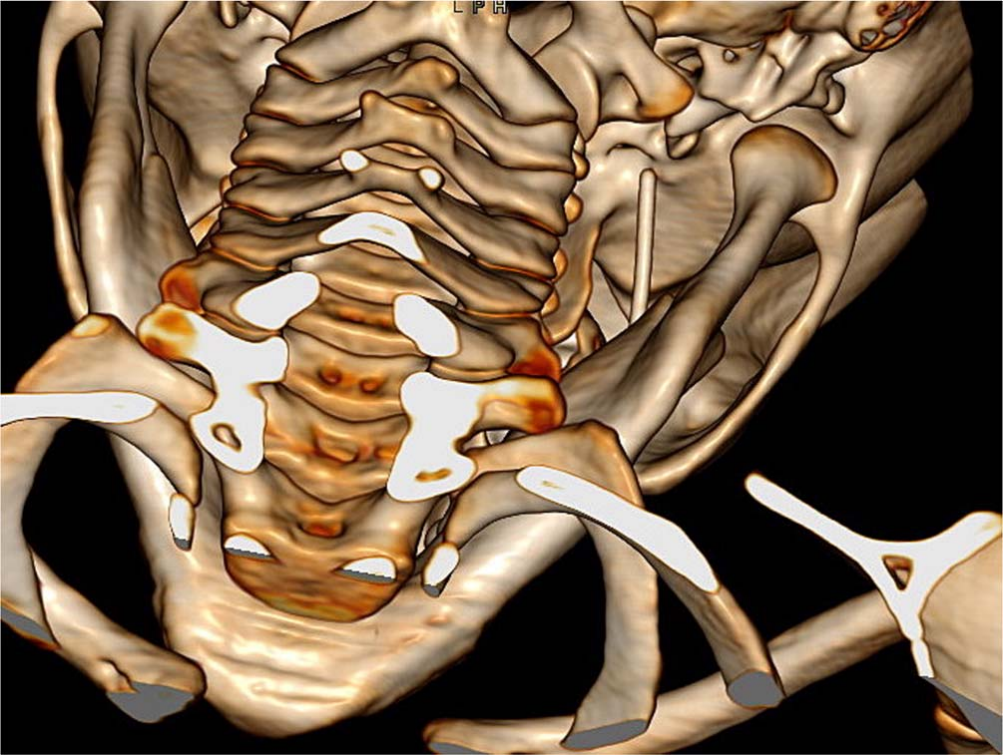
Diagnostic 3D CT showing the needle in the right pterygomandibular space.

A 4-year-old girl underwent a dental filling procedure to treat a cavity in her right maxillary molar. While blocking the posterior superior alveolar nerve, the distal end of a 31-gauge injection needle broke into her pterygoid musculature. The tip of the needle was not visible, and the dentist could not retrieve it. The patient was immediately brought to the emergency department of our centre, a tertiary referral paediatric hospital. Since the nerve block was effective, the child did not experience any pain. A diagnostic CT scan revealed the needle deep in the pterygomandibular space (Fig. 1), so surgical intervention was scheduled under general anaesthesia in an operating theatre equipped with a low-radiation C-arm cone beam computed tomography (CBCT).

**Figure 2:**
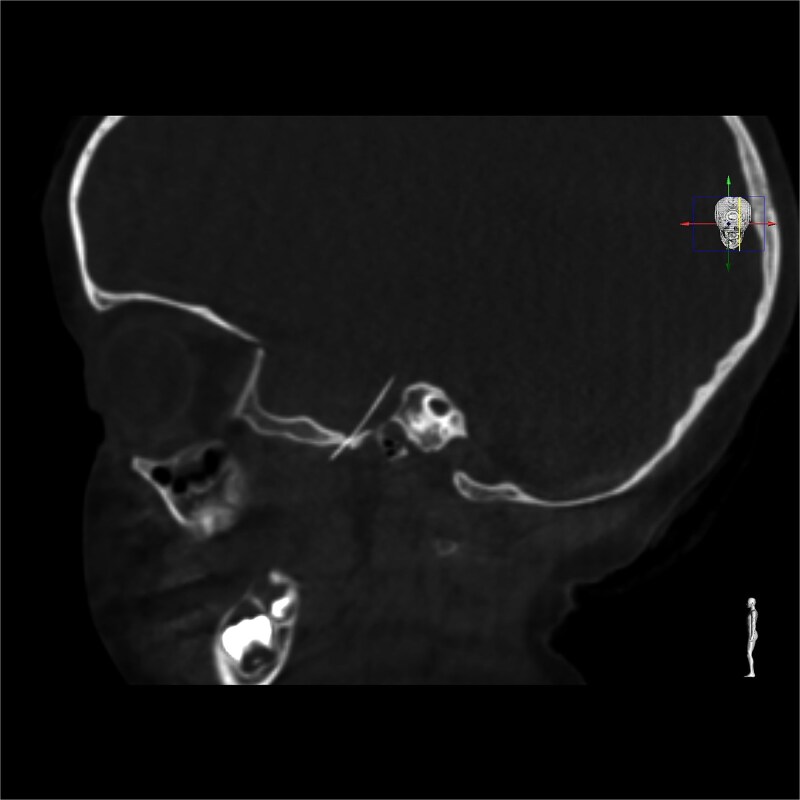
Intracranial CT scan showing the intracranial migration of the needle through the foramen ovale.

Once in the operating theatre, a CBCT was performed before starting the procedure to assess the precise position of the needle. Surprisingly, the needle had migrated, likely due to masticatory function, and had traversed the foramen ovale into the middle cranial fossa, below the right temporal lobe (Fig. 2). A neurological re-evaluation reported no neurological deficits. Given the new location of the needle, the surgical plan was revised in consultation with neurosurgeons, and the parents provided consent for a neurosurgical approach. It was agreed to perform intraoperative angiography and angio-CT to assess the integrity of the Circle of Willis.

**Figure 3:**
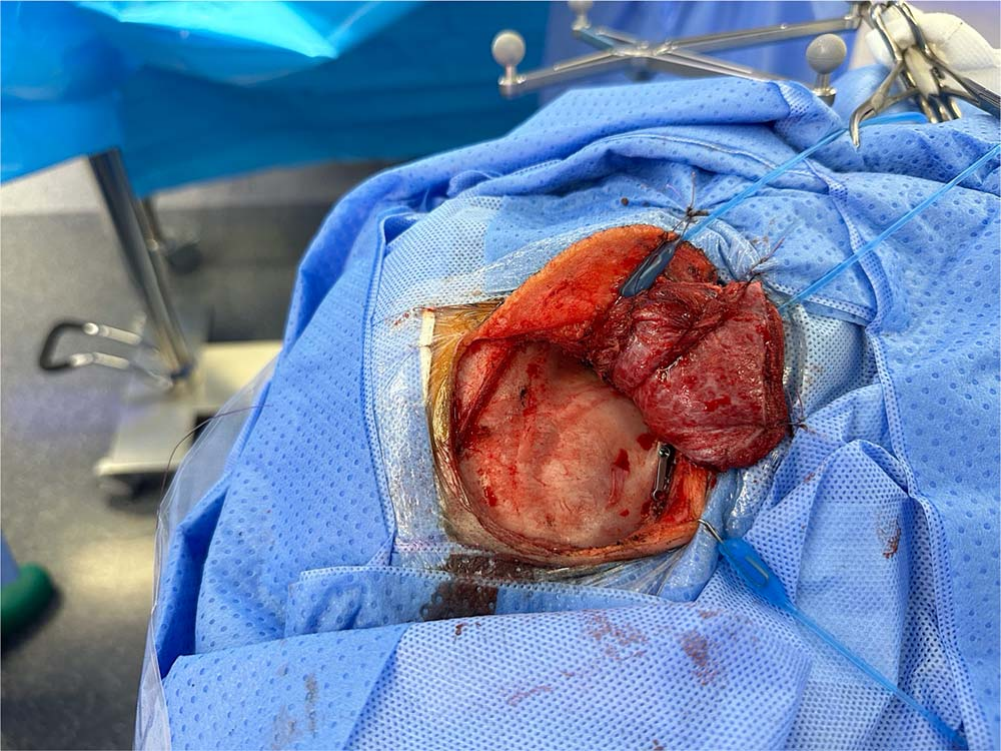
Pterional approach to reach the needle guided by neuronavigation.

Neuronavigation-assisted surgery was then performed. The patient underwent a right pterional craniotomy (Fig. 3). Extradural dissection was conducted until Meckel’s cavum. The dura was carefully opened directly over the needle, which was completely removed through the craniotomy to avoid injury to the temporal lobe. The patient was admitted for a week of post-operative monitoring and received standard prophylactic intravenous medication. After 8 days, she had recovered well and was discharged. The patient remained asymptomatic throughout the entire process.

## Discussion

Despite its rarity, needle fracture can unfortunately occur even when all preventive measures are taken. In the past, it was more common due to the use of reusable dental needles and fatigue fractures. Nowadays, needle choice, technique deficiencies, and patient management (due to sudden, abrupt movement, or swallowing) appear to be the main causes of needle breakage. Recent guidelines emphasize that thorough, safe technical implementation is the most important preventive measure [6]. This includes selecting a sufficiently long (e.g. 35 mm for IAN blocks) and strong needle (25–27 gauge), avoiding bending or kinking of the cannula, not inserting the needle into the mucosa up to the plastic hub (at least 5 mm of the needle should remain above the mucosa level) and informing the patient of what to expect and emphasizing the importance of their cooperation [3]^.^ Thus, additional care must be taken in paediatric patients and limit the use of very posterior nerve blocs to procedures affecting several teeth.

When dealing with a broken needle, in addition to an initial diagnostic CT, it is of utmost importance to have access to intraoperative imaging tools (preferably a low-radiation CT in paediatric populations) to monitor the potential risk of needle migration before or during the surgical procedure for needle removal [1].

It is important to remark that, as the patient remained asymptomatic during the whole process, a thorough multidisciplinary assessment was carried out before indicating the surgery and the potential risks that could entail. Due to the delicate location of the needle (directly pointing the temporal lobe and in vicinity of the Circle of Willis), awaiting for the appearance of clinical signs or symptoms or endangering the patient to silent injuries, was judged argument enough to carry out the intervention.

In conclusion, this report demonstrates that a broken needle may migrate intracranially, among other complications, underscoring the importance of managing these cases in a tertiary referral hospital with the appropriate equipment and specialists.

### Bullet Points

- A thorough, safe technical implementation is the most important preventive measure to avoid needle breakage: choosing the right needle, ensuring patient cooperation, and employing good handling techniques.
- If the fractured needle cannot be removed in the dental office, refer the patient to a tertiary hospital with the proper equipment and specialists.
- Due to the potential risk of needle migration, three-dimensional imaging techniques are considered the gold standard for locating foreign bodies (such as broken needles) and planning surgical interventions.
